# Transition to international classification of disease version 10, clinical modification: the impact on internal medicine and internal medicine subspecialties

**DOI:** 10.1186/s12913-018-3110-1

**Published:** 2018-05-04

**Authors:** Rachel N. Caskey, Angelos Abutahoun, Anne Polick, Michelle Barnes, Pavan Srivastava, Andrew D. Boyd

**Affiliations:** 1 Department of Internal Medicine, University of Illinois at Chicago, 840 S. Wood St, Clinical Sciences North 440, MC 718, Chicago IL, Chicago, Illinois 60612 USA; 2 Department of Pediatrics, University of Illinois at Chicago, 840 S. Wood St Clinical Sciences North 440, MC 718, Chicago IL, Chicago, Illinois 60612 USA; 3 Department of Biomedical and Health Information Sciences, University of Illinois at Chicago, 1919 W Taylor St (M/C 530), Chicago, IL, Chicago, Illinois 60612 USA

**Keywords:** ICD-10-CM, Diagnosis coding, Cohort identification

## Abstract

**Background:**

The US health care system uses diagnostic codes for billing and reimbursement as well as quality assessment and measuring clinical outcomes. The US transitioned to the International Classification of Diseases, 10th Revision, Clinical Modification (ICD-10-CM) on October, 2015. Little is known about the impact of ICD-10-CM on internal medicine and medicine subspecialists.

**Methods:**

We used a state-wide data set from Illinois Medicaid specified for Internal Medicine providers and subspecialists. A total of 3191 ICD-9-CM codes were used for 51,078 patient encounters, for a total cost of US $26,022,022 for all internal medicine. We categorized all of the ICD-9-CM codes based on the complexity of mapping to ICD-10-CM as codes with complex mapping could result in billing or administrative errors during the transition. Codes found to have complex mapping and frequently used codes (*n* = 295) were analyzed for clinical accuracy of mapping to ICD-10-CM. Each subspecialty was analyzed for complexity of codes used and proportion of reimbursement associated with complex codes.

**Results:**

Twenty-five percent of internal medicine codes have convoluted mapping to ICD-10-CM, which represent 22% of Illinois Medicaid patients, and 30% of reimbursements. Rheumatology and Endocrinology had the greatest proportion of visits and reimbursement associated with complex codes. We found 14.5% of ICD-9-CM codes used by internists, when mapped to ICD-10-CM, resulted in potential clinical inaccuracies.

**Conclusions:**

We identified that 43% of diagnostic codes evaluated and used by internists and that account for 14% of internal medicine reimbursements are associated with codes which could result in administrative errors.

**Electronic supplementary material:**

The online version of this article (10.1186/s12913-018-3110-1) contains supplementary material, which is available to authorized users.

## Background

Within the U.S. health care system diagnostic codes are used not only for billing and reimbursement, but also provide an important mechanism for monitoring health care trends and performance such as quality of care, disease prevalence, mortality and resource utilization. [1, 2] On October 1, 2015, the current system of coding medical data in the United States, the International Classification of Diseases, 9th Revision, Clinical Modification (ICD-9-CM), was replaced by the International Classification of Diseases, 10th Revision, Clinical Modification (ICD-10-CM). [1] The ICD-9-CM contains just under 14,000 codes whereas ICD-10-CM contains approximately 69,000 codes, and the codes contain much more detail compared to ICD-9-CM. [1] Compared to ICD-9-CM, the ICD-10-CM codes more accurately code ambulatory and preventive care encounters, and as health care evolves, ICD-10-CM is more flexible and has the capacity to accommodate new codes over time. [3] The International Classification of Disease (ICD) was originally developed by the world health organization to help classify and organize morbidity and mortality between countries. The ICD-9 version was modified by the U.S. government to create the Clinical Modification, (ICD-9-CM) which included more codes relevant to day-to-day clinical management of disease. The WHO revised their coding system to version 10 (ICD-10) in the 1990’s and increased the number of codes, as well as the overall organization of codes. For example, in version 9 there were 19 categories, and in version 10 there are 22 higher order categories (some new categories and some of the prior categories were divided. Multiple countries came up with different versions of ICD-10 for clinical purposes including Canada (ICD-10-CA), Germany (ICD-10-GM), Netherlands (ICD-10-nl), Australia (ICD-10-AM). In 1997, the U.S. created the U.S. government modification of the ICD-10 called ICD-10-CM with over 80,000 codes. ICD-10-CM has more detail than any other country modification in the world.

The costs associated with making the transition to ICD-10-CM in the U.S. are substantial. The transition. is estimated to cost between 475 million and 1.5 billion dollars which includes implementation of the new codes, training the medical community on use of the new codes, and adjudicating discrepancies between the two coding nomenclatures. [3] However, the amount of cost associated with transitioning to ICD-10-CM varies substantially depending on the particular medical specialty analyzed. [4] Prior analysis of the transition to ICD-10-CM showed 26% of pediatric codes, 31% of emergency medicine codes and 18% of oncology codes are complex and may lead to financial disruption. [4, 5] The objective of this study was to examine how the transition to ICD-10-CM may result in ambiguity of clinical information and financial disruption for internists and medicine subspecialists.

## Methods

### Data set

We used the Illinois Medicaid statewide dataset of ICD-9-CM data for one calendar year (2010) for all patients identified as primary care patients of the University of Illinois Hospital and Health Sciences System in the month of April 2011, as previously described. [6] All physician bills were labeled by medical specialty and sub-specialty by Medicaid, which included the amount reimbursed for each claim. The dataset was filtered for bills submitted by an internist including general internists and internal medicine subspecialists. A total of 17 internal medicine subspecialty categories from Medicaid were used (Table 1). The dataset included 3191 ICD-9-CM codes used by internists during a total of 51,078 patient encounters. Illinois Medicaid payments for these codes totaled $26,022,022. The ICD-9-CM code 999.99 [Other and unspecified complications of medical care, not elsewhere classified] was excluded from analysis as no specific diagnosis can be associated with this code. The study was approved by the University of Illinois Institutional Review Board.

**Table 1:** Categories of Physician Specialties as Labeled by Medicaid

Allergy	
Allergy-Immunization	
Cardiology	
Cardiovascular Diseases	
Endocrinology	
Gastroenterology	
General Hospital - Adults Section	
General Practice	
Geriatrics	
Hematology	
Immunology	
Infectious Diseases	
Internal Medicine	
Nephrology	
Oncology	
Pulmonary Diseases	
Rheumatology	

### Mapping ICD-9-CM to ICD-10-CM codes

The Centers for Medicare and Medicaid services (CMS) and the Centers for Disease Control and Prevention created the General Equivalent Mappings (GEMs). [1] GEMs is a mapping tool to assist with the conversion between ICD-9-CM and ICD-10-CM. The CMS General Equivalent Mappings (GEMs) has one file for directional mapping of codes from ICD-9-CM to ICD-10-CM, and a separate file for ICD-10-CM to ICD-9-CM. These files were used to create an online analysis tool (version 1.0) which was used to map the internal medicine billing codes for this project. [6]

### Categorization of complexity of mapping

**Fig. 1:**
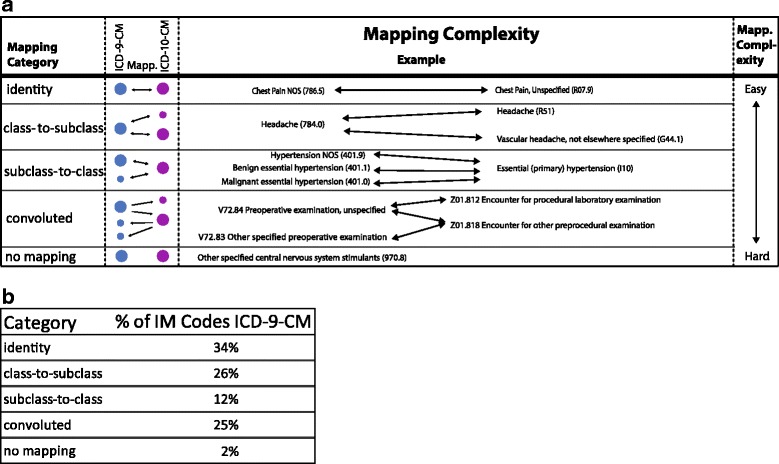
Example of the five categories of coding complexity

All of the ICD-9-CM codes have been previously categorized based on their complexity of the mapping to ICD-10-CM codes. [6] Codes were initially categorized into 28 motifs based on their mapping and these motifs were then categorized into 5 overarching categories based on the complexity of mapping between coding systems. As described previously, the codes were cataloged into five categories: *identity* (where the codes are equivalent*), class-to-subclass* (one ICD-9-CM code going to multiple ICD-10-CM codes), *subclass-to-class* (multiple ICD-9-CM codes to one ICD-10-CM code), *no transition* (no mapping to ICD-10-CM), and *convoluted* (a complex mapping of ICD-9-CM to and from ICD-10-CM (Fig. 1). [6] The first four categories describe transitions which are generally straightforward and intuitive; however, the fifth category (*convoluted*) are codes with complex mappings making the transition between the codes challenging. The internal medicine ICD-9-CM codes were mapped to ICD-10-CM codes and were then designated into one of the above five categories. A Cohen’s Kappa was calculated to compare coding between physicians.

### Data analysis

The internal medicine ICD-9-CM diagnosis codes, patient visit count and cost analysis was performed for each of the five categories of codes. The internal medicine ICD-9-CM dataset was analyzed by subspecialty to determine the percentage of codes by category, the percentage of visits by code category (based on codes billed by visit) and the percentage of reimbursed dollars by code category.

To understand the potential challenges inherent in the transition to ICD-10-CM, the frequently used *convoluted* ICD-9-CM codes, defined as codes used 10 or more times in the dataset (10 or more bills submitted with the code), and the high utilization codes, defined as the 20 most frequently used codes in the non-complex mapping categories (*identity*, *class-to-subclass, subclass-to-class),* were identified. The *no mapping* category was excluded as there were very few codes in this category. The codes were evaluated by practicing internal medicine physicians and classified into one of four previously defined categories: information loss, overlapping categories, inconsistent or consistent. [4] “Information loss” was used when the transition to ICD-10-CM obscures a clinically important diagnostic distinction, resulting in a potential loss of relevant clinical detail. “Overlapping category” was used when the transition results in a distinction without a clinically relevant difference or results in confusion regarding appropriate code assignment. The overlapping category included ICD-9-CM codes which mapped to ICD-10-CM codes where additional non-intuitive clinical information was needed to understand the mapping. “Inconsistent” was used when an ICD-10-CM code was clinically different than the ICD-9-CM code to which it mapped. “Consistent” category was used when the transition resulted in a consistent and clinically intuitive ICD-10-CM code (see Table 2). [4]

**Table 2:** Category Examples for ICD-9-CM Codes based on Transitions to ICD-10-CM Codes

Category	ICD9 Code	ICD10 Code(s)	Description
Information Loss	413.0 Angina Decubitus	I20.8 Other forms of angina pectoris	ICD-10-CM code loses the designation of *decubitus* which clinically provides context for the type of angina (while supine)
Overlapping Categories	272.4 Other and unspecified hyperlipidemia	E78.5 Hyperlipidemia, unspecified E78.4 Other hyperlipidemia	The ICD-9-CM maps to two ICD-10-CM codes, it is not apparent to the practicing physician the difference between these two ICD-10-CM codes
Inconsistent	719.49 Pain in join involving multiple sites	M25.50 Pain in unspecified joint	719.49 is designated as as multiple joints but M25.50 is designated as a single joint

Four practicing internists (RC, MB, PS, AP) conducted the assignment of diagnosis codes into the four categories. At least two independent physicians analyzed and classified each code and all discrepancies in classification were reviewed and rectified by the group. When analyzing the mappings the physicians focused on clinical accuracy and clinical relevance of each mapping. Because more experienced physicians may have coding practices which have been influenced by reimbursement rates or patient payer mix over the years we selected two junior physicians (less than four years since residency completion) and two mid-career (7–10 years since residency completion) physicians to conduct the coding. None of the physicians have billing expertise outside of on-the-job experience as a practicing internist.

To calculate the financial impact of the different ICD-9-CM categories, the labeled ICD-9-CM codes were associated with the number of visits and payment from the Illinois Medicaid database billed by internal medicine physicians.

## Results

Of the 3191 ICD-9-CM codes used by internists, 25% of the primary diagnosis codes were convoluted and 22% of the patient visits were convoluted. Also, 30% of the total payment Internal medicine physicians had convoluted codes associated with them (Table 3). A total of 52 ICD-9-CM codes had no ICD-10-CM (1.6% of unique codes) mapping.

**Table 3:** Percentage and numbers of Patient Visits and Reimbursement in Illinois Medicaid related to Internal Medicine and all subspecialties

	Patient Visits	Medicaid Reimbursement	ICD-9-CM codes
Identity	35% (17,961)	32% ($8,208,151)	34% (819)
Class to subclass	27% (13,803)	23% ($5,973,213)	26% (644)
Subclass to class	15% (7501)	15% ($3,811,169)	12% (300)
Convoluted	22% (11,217)	30% ($7,881,304)	25% (616)
No translation	1% (580)	1% ($145,845)	2% (50)

**Fig. 2:**
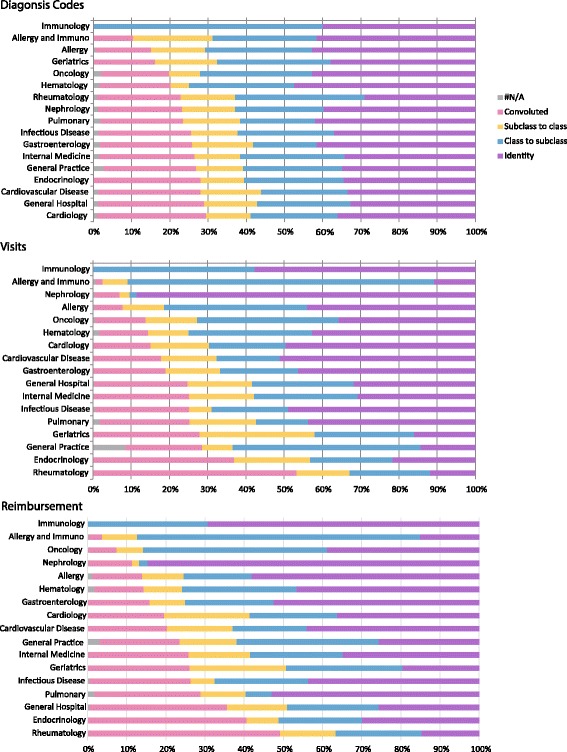
Analysis of Internal Medicine specialty for codes, visits, and reimbursement

An analysis of each subspecialty found that general medicine and general hospital medicine, as well as some subspecialties, used the greatest percentage of convoluted codes. General medicine, endocrinology and rheumatology had the greatest number of visits associated with a convoluted code, with over 50% of rheumatology visits billed with a convoluted code (Fig. 2). The reimbursement by subspecialty resulted in a similar pattern - general medicine, endocrinology and rheumatology had the largest percentage of reimbursements associated with convoluted codes.

We evaluated 295 codes which included 235 frequently used *convoluted* ICD-9-CM codes and 60 high utilization codes. The physician coders Cohen’s kappa coefficient was initially 0.55 and the coders reviewed every discrepancy and consensus was reached on the classification for every code. Of the codes with disagreement, the majority of discrepancies were due to initial differences in clinical interpretation of the codes. A kappa was repeated after initial consensus on the codes was reached and increased to 0.89. Of the 295 codes evaluated, 43% were coded as information loss, overlapping, or inconsistent (Table 4, Additional file 1 Table S1 for specific codes in Supplementary File). Payments for these three categories of codes accounted for 14.5% of all reimbursements statewide; totaling $160,541 for information loss, $2,718,281 for overlapping, and $920,140 for inconsistent (Table 4). Code 999.99, which was excluded from our analysis, was used in 73,709 visits, for a cost of $214,603.

**Table 4:** Analyzed Internal Medicine ICD-9-CM Codes out of all ICD-9-CM Codes in Illinois Medicaid

	Number of analyzed Codes (*N* = 295)	Percent of total IL Medicaid diagnosis codes	Reimbursement (dollars) and percent of total IL Medicaid Reimbursement
Information Loss	36 (12%)	1.1	$160,541 (0.6%)
Overlapping categories	54 (18%)	1.7	$2,718,281 (10.4%)
Inconsistent	39 (13%)	1.2	$920,140 (3.5%).
Consistent	166 (56%)	5.2	$7,875,660 (30% of total cost)

## Discussion

The transition to ICD-10-CM is a substantial administrative change occurring at a time of rapid transformation of the U.S. health care system. Diagnostic code data are used broadly for billing and tracking purposes making the potential impact of this transition to ICD-10-CM sizable. Diagnostic codes are used to indicate a diagnosis associated with a health care encounter and to determine medical necessity for treatment. We have highlighted the codes with convoluted mapping and the potential for mapping errors as these are the codes which are likely to result in billing and data tracking errors. We found 14% of reimbursement dollars received by internal medicine physicians in 2010 were associated with codes categorized as *information loss, overlapping categories* or *inconsistent.* In our analysis, 43% of the codes had changes in information between ICD-9-CM and ICD-10-CM (information loss, overlapping categories, and inconsistent). Our team only evaluated the most complex and frequently used codes and evaluated only 44% of the reimbursement related codes for internal medicine. There is the potential for financial disruption due to inaccurate mappings to ICD-10-CM, often referred to as the revenue gap of ICD-10-CM. [7] For example, after completing an ambulatory visit for management of hyperlipidemia, which historically could have been coded as ICD-9-CM 272.4 (*Other and unspecified hyperlipidemia*), a clinician could now select either E78.5 (*Hyperlipidemia unspecified*) or E78.4 (*Other hyperlipidemia*). The decision of which code to select may seem arbitrary but the reimbursement may differ as one code may be a higher reimbursed service by a payer despite the fact there is no intuitive difference to the billing clinician. Further, if the clinician wanted to later identify all patients with hyperlipidemia the clinician would need to search for patients with either E75.8 or E78.4 diagnostic codes as both are needed to identify patients with this diagnosis.

Data tracking becomes even more cumbersome for codes which do not have bi-directional mapping. For example, GEMS files are the accepted standard for mapping between the two sets of codes. However, when mapping from ICD-10-CM to ICD-9-CM GEMS maps to only 70% of ICD-9-CM codes and when mapping from ICD-9-CM to ICD-10-CM GEMS maps to only 24% of ICD-10-CM codes. A large number of codes are missed using this CMS provided mapping tool. Further, this transition will have an impact on prospective and retrospective research trials which use diagnostic codes. Comparing diagnostic codes before and after the transition to ICD-10-CM will be difficult and future studies will need an explicit description about how the mapping between ICD-9-CM and ICD-10-CM was conducted. Additional mappings models have been provided by both Federal and commercial providers; including the CMS reimbursement mapping. We chose not to use the CMS reimbursement mapping method as this mapping was only updated in 2015 and 2016 and the GEMs files have been updated annually since 2011.

The complexity of the transition to ICD-10-CM is multifactorial. The WHO changed the overall organization and structure of the ICD codes for ICD-10-CM. Individual countries then modify the codes for use in their health care system, which adds another layer of complexity and limits comparisons between countries. For example, in the U.S. obstetric codes are required to label a pregnancy visit as first trimester, second trimester, third trimester or unspecified trimester (represented in the final digit of 1,2,3, or 9 respectively). This obstetric coding structure is not used in other countries. While the new coding may lead to a more precise understanding of clinical care provided or patient characteristics, the complexity of ICD-10-CM limits use of the codes in research. In addition, the code complexity varies by field. Hematology has less than 10% convoluted codes compared to Obstetrics where over 60% of codes are convoluted. [6]

This is the first analysis of internal medicine subspecialties, all of which will be impacted by the transition to ICD-10-CM. Among the internal medicine fields we found general medicine, endocrinology and rheumatology had the largest percentage of visits associated with convoluted (complex) codes and/or no translation. No translation is a troubling category as there is no method to track or report these codes across the transition. Further, hospital medicine, endocrinology and rheumatology had the largest percentage of reimbursements associated with convoluted codes, higher than other medical fields including pediatrics and emergency medicine. [8] The potential financial disruption will likely not be felt equally across internal medicine subspecialties suggesting more resources should be dedicated to those fields that are at greatest risk for financial disruption by the transition and impact into clinical research. Recent news articles have shown the level of financial disruption to clinics, where 18% of providers have a higher denial rate after the transition. [9] Due to the expected challenges during the transition to ICD-10-CM the Center for Medicare and Medicaid Services (CMS) announced that claims will not be denied due to lack of coding specificity, and CMS will work to minimize payment disruptions, for the first 12 months. [10] This concession by CMS likely alleviated some anxiety during the transition; however, now reimbursement will be tied to the level of specificity of the codes. In addition, due to this concession the specificity of the ICD-10-CM codes from October, 2015 to October, 2016 will be highly variable making future comparisons of code data difficult.

One unexpected finding was the level of initial disagreement between the physician coders. In prior work by the authors 20% disagreement between coders was found. [4] Internal medicine has a larger number of convoluted codes compared to other fields which likely attributed to the difference. In addition, training of physicians about ICD-10-CM has largely focused on learning the new codes and the associated changes in documentation which will be required, rather than strategies for selecting the optimal code. [11] In practice, many clinicians may select the first ‘reasonable’ code provided by the electronic medical record, even if the code is not the most specific. Given the complexity of codes we identified in internal medicine, particularly among some subspecialties, efforts are needed to carefully monitor the impact from the transition to ICD-10-CM and develop strategies for clinicians to easily identify and select optimal codes.

### Limitations

Our classifications of codes will not impact daily clinical practice. However, when longitudinally comparing outcomes (quality improvement, care outcomes, costs) these issues related to transition to ICD-10-CM are important. The Illinois Medicaid database is limited to the physicians who accept IL Medicaid. Physicians who deliberately select private insurance and/or Medicare could have a different coding practice, as well as a different disease burden among their patients, compared to those who accept Medicaid. Medicaid reimburses less than many insurers, so the financial impact to health care is likely underestimated by this analysis. Physicians are able to assign multiple ICD codes to a single encounter, in addition, the structure of ICD-10-CM requires secondary codes for some conditions (ex; overdose). For the purpose of this analysis we focused only on the primary codes, and we did not include secondary codes in the analysis. Finally, the interpretation of codes and definitions may be impacted by training, experience, and biases.

## Conclusion

We identified that 43% of diagnostic codes commonly used by internists and 14% of internal medicine reimbursements are associated with codes which could result in financial disruption and administrative errors for internists and subspecialists. Sub-specialties are likely to be impacted differently, with rheumatology having the most challenging transition with up to 50% of all office visits associated with codes that have complex mapping. Additional research is needed into the appropriate training and method to help assign accurate codes by both providers and professional coders. Improving current mapping tools (including GEMS) will be critical for a smooth transition to ICD-10-CM for clinicians and to permit accurate data for researchers and policy makers who will no doubt be studying the impact on outcomes for many years to come.

## Additional file

Additional file 1: **Table S1**. Categorization of ICD-9-CM codes by Information Loss, Overlapping, and Inconsistent. The data is a concatenated list of ICD-9-CM codes without the decimal into three lists of information loss, overlapping and inconsistent. The ICD-9-CM codes in these lists need additional work before use in evaluation of data during the transition from ICD-9-CM to ICD-10-CM. (DOCX 13 kb)

## Abbreviations

CMS: Center for Medicaid and Medicare Services
GEMs: General Equivalent Mappings
ICD-10-CM: International classification of Disease Clinical Modification version 10
ICD-9-CM: International classification of Disease Clinical Modification version 9
U.S: United States
