# Supplementary material for: Transition to international classification of disease version 10, clinical modification: the impact on internal medicine and internal medicine subspecialties
Source: BMC Health Serv Res. 2018 May 4;18:328. doi: 10.1186/s12913-018-3110-1 (PMC5935982; doi:10.1186/s12913-018-3110-1)
Supplement: Supplementary file 1 — Table S1. Categorization of ICD-9-CM codes by Information Loss, Overlapping, and Inconsistent. The data is a concatenated list of ICD-9-CM codes without the decimal into three lists of information loss, overlapping and inconsistent. The ICD-9-CM codes in these lists need additional work before use in evaluation of data during the transition from ICD-9-CM to ICD-10-CM. (DOCX 13 kb) [file 12913_2018_3110_MOESM1_ESM.docx]

Supplementary Table 1

| Information loss | Overlapping | Inconsistent |
| --- | --- | --- |
| 4130 | 5601 | 2808 |
| 25062 | 6259 | 4539 |
| 25063 | 6269 | 6820 |
| 72402 | 25013 | 6823 |
| V063 | 25082 | 6961 |
| 2967 | 61610 | 25012 |
| 42781 | 71944 | 7103 |
| 07054 | 78099 | 64891 |
| 86121 | V679 | 4259 |
| V032 | 4254 | 4430 |
| V0381 | 6268 | 7964 |
| V0389 | 7810 | 8798 |
| V0489 | 1179 | 41401 |
| V054 | 1977 | 49300 |
| V064 | 2724 | 64683 |
| 436 | 3572 | 68101 |
| 2801 | 4148 | 70715 |
| 04185 | 5718 | 71949 |
| 5173 | 7101 | 73008 |
| 9583 | 9961 | 73349 |
| 25010 | 25080 | V702 |
| 37993 | 51881 | V717 |
| 71695 | 71500 | 5550 |
| 73027 | 71590 | 5559 |
| V0382 | 78799 | 6824 |
| V040 | 99859 | 6826 |
| V0481 | 1120 | 6827 |
| V053 | 2409 | 7108 |
| V061 | 3009 | 8479 |
| V065 | 4139 | 29980 |
| V069 | 4660 | 30590 |
| V0731 | 5768 | 41070 |
| V151 | 6089 | 56211 |
| V1581 | 7840 | 68100 |
| V481 | 7906 | 76510 |
| V711 | 7919 | 78079 |
|  | 7932 | 78909 |
|  | 20280 | V290 |
|  | 30742 | V6759 |
|  | 34550 |  |
|  | 41090 |  |
|  | 42789 |  |
|  | 64663 |  |
|  | 64893 |  |
|  | 71101 |  |
|  | 78060 |  |
|  | 78609 |  |
|  | 78659 |  |
|  | 78703 |  |
|  | 78791 |  |
|  | 79099 |  |
|  | 79579 |  |
|  | V705 |  |
|  | V7284 |  |
